# University of London—Medical Diploma

**Published:** 1830-07-01

**Authors:** 


### XXII.

### University of London.

### *Medical Diploma.*

"In the 'Second Statement by the Council,' published before the opening of the University in 1828, it is announced that '*besides Certificates of the Professors, the University will grant Certificates of General Proficiency in Literature and Science. Every Student will be required to produce a certain number of Professors Certificates, before he can be allowed to enter upon the examination for the General Certificate.*"

The object in granting this Certificate, is to put the Student in possession of a document which shall be an evidence of his having acquired at the

University a certain amount of knowledge in the different departments of General and Professional Education. But to make the document practically useful, it must have such *a designation* as the person obtaining it can conveniently affix to his name and be called by; as is the case when he takes a Degree at an Incorporated University.

The Council have been for some time engaged in considering the Qualifications, Conditions, and the Form under which this Diploma shall be granted in the different departments of education; they have not yet settled what these shall be in any other than the Medical School, but expect to be able to announce the whole scheme before the conclusion of the present Session. Having come to a decision upon the Medical Diploma, the Council have thought it advisable to make it known before the Medical Students of the present Session shall be dispersed.

It has been resolved that the General University Certificate shall be granted, in the Medical School, under the following conditions and regulations, and that it shall be called 'The Diploma of Master of Medicine and Surgery in the University of London,' (which may be thus translated and abbreviated, *M. Med. et Chir. U. L.*).

1. That the Candidate shall be Twenty-one Years of age.

2. That he shall have attended Lectures on Professional subjects, during three Academical Sessions of this University, and one winter Session of at least five months duration in any established school at home or abroad.

3. That he shall have acquired Certificates of Honor* in the following Classes in the University:

* "It is proper to explain to those unacquainted with the system of the University, that Certificates of Honour are granted at the conclusion of each Session to the more distinguished Students in the several Classes. The distinction is conferred, if answers in writing to questions proposed during a very carefully conducted examination, shall prove that the Student must have diligently attended to the instruction of the Professor."

Practice of Medicine,—Anatomy,—Physiology,—Surgery,—Midwifery, and Diseases of Women and Children,—Materia Medica,—Botany,—Chemistry,—and Anatomical Demonstrations and Dissections.

4. That in the year which may be spent out of the University, he shall have attended Lectures on two different professional subjects, each Course of such Lectures being of at least five months duration.

5. That he shall have attended the Medical Practice of an Hospital, containing at least 100 beds, for twelve months; and the Surgical Practice of the same Hospital, or another Hospital of the same number of beds, for the like period of twelve months.

6. That he shall be required to translate a passage in writing from Celsus, Gregory, Heberden, or other Latin medical author.

7. That having complied with the preceding regulations, he shall write an Essay in the English language on some professional subject, chosen by himself, and approved of by the Faculty of Medicine before the Essay is composed. That this Essay shall be read in whole or in part, as the Faculty may desire, at a public meeting in the University; and that the Candidate shall be called upon to explain or defend the doctrines maintained in his Essay. That he shall also make an Anatomical Demonstration, and be examined upon any part of his professional studies on which the Faculty of Medicine may think proper to propose questions.

---

In proposing the above regulations for conferring an honorary distinction, suited to Surgeons and General Practitioners, the Council have thought it proper to require attendance on those Classes only which are necessary for obtaining the Diploma of the College of Surgeons of London, and Society of Apothecaries. But they are desirous that the attention of Medical Students should also be particularly directed to the subjects of Clinical Medicine, Comparative Anatomy, and Medical Jurisprudence: and it will also be a great recommendation to candidates that they should possess some knowledge of MATHEMATICS, NATURAL PHILOSOPHY, and NATURAL HISTORY. The scheme of instruction in the University affords ample opportunity for such studies; and a diligent Pupil, during the period prescribed by the Regulations, may obtain respectable knowledge in several of these departments, without interfering with the more direct object of his pursuits.

The Diploma will be conferred in public on the 23rd of December in each year, or the 22nd, if the 23rd be a Sunday; and the Examinations will commence on the corresponding day of the preceding week. The Essay must be sent to the Secretary of the Faculty of Medicine on or before the 15th of November, and must be signed by the Candidate, with a declaration that it is wholly his own composition. In the event of Students leaving England, or in other cases of emergency, the Diploma will be granted at other periods of the Session, if the Candidates possess the necessary qualifications.

In selecting the designation, care has been taken to avoid all interference with the titles and privileges conferred by Chartered Bodies. The value of the Diploma to the possessor of it will depend upon its being known to be granted to those only who, after a strict examination, prove themselves worthy of such a distinction.

---

The following extract from a Report of the Faculty of Medicine, contains their views as to the good effects upon the education of Medical Students which this measure may tend to produce.

'The Medical Profession is divided into three classes: viz. Physicians, Surgeons, and General Practitioners. The latter form by far the greater body, and until this University can give a Physician's Degree, not many of those destined for that branch of the profession can be expected to take any considerable part of their education in its Medical School.

'Under the appellation of General Practitioners are included two distinct classes of medical men. One of these consists of practitioners who hold a highly respectable rank in the profession, and who have devoted much time, labour, and money to their professional education; men possessed of some attainments in the collateral sciences, and who, practising their profession in a liberal and scientific spirit, have the highest claim to the confidence of the public. Another class bearing the same appellation, consists of those who have acquired the right to practise by possessing only the *minimum* of knowledge by which the licence can be obtained, earned by the smallest possible expenditure of time and labour, and who consequently have very imperfect professional attainments. The public possess so little knowledge of the details of a Medical and Surgical Education, that all the most serious duties of the profession are commonly confided, without inquiry, to any one who calls himself a general practitioner, and to such hands, especially in the country, the largest portion of professional duty and responsibility is intrusted.

'It becomes therefore a great duty for the University to endeavour to remedy the evil, as far as it has the means of doing so, by holding out this honorary distinction as encouragement to General Practitioners to follow such a more extended course of study as the science they profess, and as the public interest require.'

(By order of the Council.)
LEONARD HORNER,
*Warden*.

*University of London*,
*24th April*, 1820."

---

XXIII.

